# A conserved KL2-capsule-related phage-resistant mechanism in carbapenem-resistant *Acinetobacter baumannii* is surmountable by a rationally selected dual-phage cocktail

**DOI:** 10.3389/fcimb.2026.1790430

**Published:** 2026-04-02

**Authors:** Yong Shao, Zhu Gao, Hongyang You, Shaokun Zhang, Jiaqi Zeng, Jiawei Zhao, Chenyan Yuan, Fengqin Miao, Yuqing Shen, Ying Zhang, Ling Liu, Jianqiong Zhang

**Affiliations:** 1 School of Life Science and Technology, Southeast University, Nanjing, China; 2 Department of Microbiology and Immunology, School of Medicine, Southeast University, Nanjing, China; 3 Department of Clinical Laboratory, Zhongda Hospital, Southeast University, Nanjing, China; 4 Jiangsu Provincial Key Laboratory of Critical Care Medicine, Department of Critical Care Medicine, Zhongda Hospital, School of Medicine, Southeast University, Nanjing, China

**Keywords:** *Acinetobacter baumannii*, bacterial infection, phage resistance, phage therapy, phage cocktail

## Abstract

**Introduction:**

As the use of bacteriophages to combat drug-resistant bacteria increases, phage resistance, which may lead to infection recurrence, has gradually gained attention. The intricate mechanisms underlying phage resistance, which are crucial for improving the efficacy of phage therapy, have not yet been fully delineated. This research focuses on uncovering the mechanisms of phage resistance and formulating robust strategy to address infections caused by carbapenem-resistant *Acinetobacter baumannii* (CRAB).

**Methods:**

A specific bacterium-phage pair (CRAB strain MRAB11 and lytic phage MRABP9) was employed. Phage-resistant strains were isolated and subjected to whole−genome sequencing, followed by comparative genomics analysis. A novel phage targeting phage-resistant strains, was screened and combined with MRABP9. The efficacy of the phage combination was validated in bacterial liquid growth and in mouse wound CRAB infection model. The antimicrobial spectra of phage MRABP9 and the phage combination were evaluated using multi-center clinical *A. baumannii* isolates, and the fitness of phage-resistant strains was determined.

**Results:**

All sequenced phage-MRABP9-resistant strains harbored mutations on KL2 locus under both *in vitro* and *in vivo* conditions. A novel phage MRABphi22 targeting phage-resistant strains was introduced and its combination with phage MRABP9 effectively inhibited the growth and phage resistance development of CRAB, demonstrating promising efficacy in mouse wound infection treatment. The fitness of phage-resistant isolates in terms of antibiotic resistance, virulence and growth rate, was diminished, with dual-phage resistant strains bearing higher fitness costs.

**Conclusions:**

The mechanism underlying the response of MRAB11 to phage MRABP9 invasion exhibits high consistency on KL2 locus and is surmountable by a well-designed dual-phage cocktail. This study offers valuable guidance for future experimental design of clinical phage therapy, and points out potential therapeutic phage candidates for treating CRAB infections.

## Graphical Abstract

**Figure:**
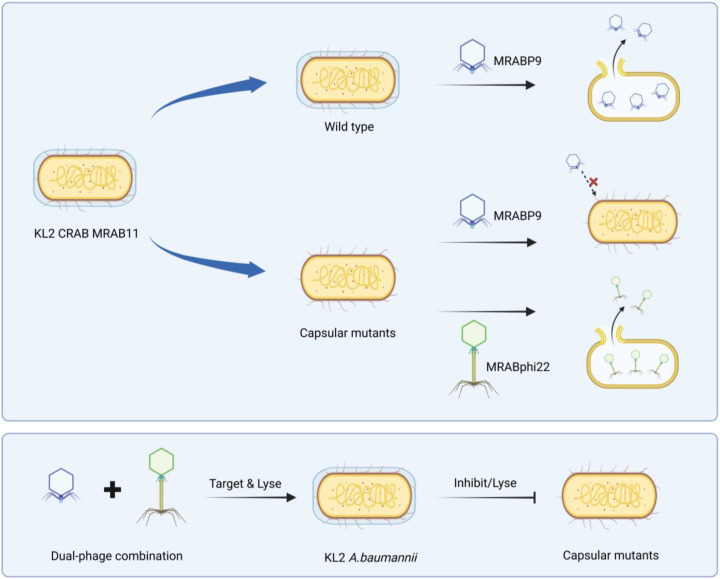
Shao, Y. (2026) https://BioRender.com/7rhrtvg.

## 1 Introduction

Bacterial infections pose a substantial threat to public health. Since the clinical introduction of penicillin, antibiotics have served as the cornerstone of infectious disease treatment. However, their widespread and often inappropriate use has driven the dissemination of resistance genes across clinical, agricultural, and environmental settings, thereby facilitating the emergence of multidrug-resistant (MDR) bacteria (Borin et al., 2021; Davies and Davies, 2010; Zhang et al., 2009). MDR bacteria are projected to cause approximately 8.22 million deaths annually by 2050 (Collaborators, 2024). Among these pathogens, *Acinetobacter baumannii*— a typical MDR Gram-negative bacterium— is a clinically prominent nosocomial pathogen associated with severe infections including meningitis, pneumonia, urinary tract infections, and sepsis, especially in immunocompromised individuals (Wang et al., 2022a). *A. baumannii* readily forms robust biofilm and acquires resistance genes via horizontal gene transfer, two traits that severely compromise antibiotic efficacy (Gedefie et al., 2021; Kaushik et al., 2022; Thummeepak et al., 2016) and drive the persistence and global spread of multidrug-resistant strains (Huang et al., 2013; Kyriakidis et al., 2021; Liu et al., 2022). As the last line of defense for severe infections (Meletis, 2016; Thacharodi et al., 2024), carbapenems are increasingly ineffective due to the alarming rise in carbapenem-resistant *Acinetobacter baumannii* (CRAB) (Adeyemi et al., 2025; Itani et al., 2023; Nowak et al., 2017). Recognized by the World Health Organization as a critical priority pathogen (Raza et al., 2021; Sati et al., 2025; Tacconelli et al., 2018; World Health Organization, 2024), CRAB underscores the urgent need for novel therapies.

In response to this crisis, bacteriophage therapy has re−emerged as a highly promising antimicrobial approach with distinct advantages over traditional antibiotics: strict host specificity, the ability to target only pathogenic bacteria without disrupting commensal microflora (Abd El-Aziz et al., 2019; Agarwal et al., 2018), and self-replication at the infection site to achieve sustained antibacterial efficacy (Agarwal et al., 2018). Moreover, certain phages can effectively disrupt bacterial biofilms and increase the susceptibility of drug-resistant bacteria to antibiotics, thus enabling their combined use with antibiotics (Abd El-Aziz et al., 2019; Luo et al., 2022). However, individual phages generally infect only a limited number of bacterial strains or even a single serotype. This narrow host range makes it difficult to match phages with the actual pathogenic bacteria isolated from patients, and also limits the efficacy of phage therapy against polymicrobial infections which are highly prevalent in clinical settings. Current strategies often involve the use of designed phage cocktails to broaden antimicrobial spectra, and certain phage-antibiotic combinations have demonstrated synergistic effects that enhance bacterial clearance (Luo et al., 2022). Beyond host range constraints, another major challenge limiting phage therapy is the emergence of phage resistance. Phage resistance describes the phenomenon in which bacteria develop resistance to phages that were initially effective, potentially leading to treatment failure and infection relapse. However, effective strategies to overcome phage resistance remain scarce. Therefore, elucidating the mechanisms underlying phage resistance is crucial not only for enhancing the effectiveness of phage therapy but also for advancing fundamental knowledge of phage–bacteria interactions.

In this study, the clinical CRAB strain MRAB11 and its lytic phage MRABP9 were employed to investigate phage resistance. By elucidating the mechanism underlying phage resistance and identifying novel phage capable of broadly targeting resistant strains, this study aims to provide a theoretical and experimental foundation, along with promising therapeutic phage candidates, for the treatment of clinical refractory CRAB infections.

## 2 Materials and methods

### 2.1 Bacterial strains and culture conditions

A total of 111 clinical isolates of *A. baumannii* were obtained from 3 hospitals located in 3 cities across Jiangsu Province, China, including 60 isolates from Zhongda Hospital Southeast University (Nanjing), 20 from Yixing People’s Hospital (Wuxi), and 31 from the First People’s Hospital of Lianyungang (Lianyungang). Bacteria were cultured in LB broth and maintained aerobically at 37 °C. Bacterial stocks were preserved in medium supplemented with 20% (v/v) glycerol and stored at -80 °C.

### 2.2 Animals

Mice, including BALB/c and C57BL/6J strains, were purchased from Hangzhou Ziyuan Laboratory Animal Technology Co., Ltd. (China). At the end of the experiments, the mice were euthanized by CO_2_ inhalation after isoflurane anesthesia. All procedures were approved by the Animal Care and Use Committee of Southeast University (approval number: SEU-IACUC-20250912202), and complied with relevant institutional guidelines.

### 2.3 Isolation of phage-resistant bacterial strains

Phage-resistant strains isolated from liquid cultures: bacterial strain MRAB11 was cultivated to the logarithmic phase and adjusted to 1×10^7^ CFU/mL. Phage MRABP9 was added (MOI = 0.01), followed by incubation at 37 °C for 20 h. Bacterial-phage cultures were streaked and monoclonal bacterial colonies were selected. This procedure was repeated twice more to ensure complete bacterial isolation and purification.

Isolated from plate cultures: a 200 μL aliquot of logarithmic-phase bacterial culture was mixed with 50 μL of serially diluted phage suspension. The mixture was combined with 3 mL molten soft agar, poured onto a pre-warmed LB agar plate, and solidified. After incubation at 37 °C for 12 h, plates showing clear phage lysis were selected for further 36-h incubation. Individual colonies were isolated and purified by streak plating.

Isolated from mouse abdominal infection model: male C57BL/6J mice aged 10–12 weeks (28–32 g) were infected intraperitoneally with MRAB11 wild-type (WT) at a dose of 1×10^8^ CFU/20g body weight. 2 h post-infection, phage MRABP9 was administered (1×10^6^ PFU/20g body weight). After 24 h, mice were euthanized, and 2 mL of PBS was injected into the peritoneal cavity to collect peritoneal fluid. Bacteria were then isolated and purified from the recovered fluid.

Isolated from mouse wound infection model: the model was established as previously described (de Vries et al., 2020; Sweere et al., 2019) with minor modifications. Female BALB/c mice (20–25 g, 8–10 weeks) were used. Briefly, dorsal hair was removed two days before inoculation, and an 8-mm diameter circular wound was surgically created. The wound was then covered with a sterile dressing. One day following wounding, 1×10^7^ CFU bacteria were inoculated into each wound. Phage treatment (1×10^5^ PFU/wound) was administered one day after bacterial inoculation. One day thereafter, a subset of the mice was euthanized and wound tissues were collected for bacteria recovery. For treatment, phage MRABP9 and MRABphi22 were administered at 1×10^5^ PFU/wound, while the control group received an equal volume (10 μL) of sterile saline. Mouse wounds were photographed daily at the same time using a camera. The wound edges were outlined and the wound areas were quantified using Fiji (v2.16.0/1.54p) software according to the scale bar included in each photograph. All area measurements were performed independently by three investigators to minimize subjective bias. Daily data were normalized to the wound area of each mouse on day 1 post-bacterial infection (the start of treatment), which was set as 100%.

All isolates from the four sources were evaluated for phage resistance via the cross-streak method.

### 2.4 Assessing phage-resistance by cross-streak assay

This experiment was adapted from protocols previously described (Hesse et al., 2021; Prazak et al., 2019), with minor modifications: The test isolates were cultured to the logarithmic phase, with OD_600_ of approximately 0.5. Streaking the control and test bacteria in parallel lines onto an LB agar plate without cross-contamination. The plate was left undisturbed for 3–5 min to allow complete absorption of bacterial suspension. The phage suspension was prepared in advance, with the titer of 1×10^9^ PFU/mL. Using a sterile inoculation loop, the phage suspension (~2 μL) was streaked perpendicularly across the bacterial line. The cross-streak plates were incubated at 37 °C for approximately 12 h, and phage resistance was assessed the following day based on bacterial growth along the streak lines, with a complete bacterial growth band indicating phage resistance. Experiments were performed independently in triplicate.

### 2.5 Antibiotic sensitivity testing

Susceptibility to antibiotics was evaluated using the minimal inhibitory concentration (MIC) assay, performed as previously described (Belanger and Hancock, 2021). In brief, the test bacteria were cultured at 37 °C until reaching the logarithmic phase. Antimicrobial agents were prepared in Mueller-Hinton (MH) broth. Aliquots of the bacterial suspension and antimicrobial agents were then added to microplates and mixed gently, resulting in a final bacterial concentration of 10^5–^10^6^ CFU/mL. A growth control using bacterial culture and a sterility control with sterile LB medium were simultaneously established. In this experiment, the *Escherichia coli* ATCC 25922 was employed as the quality control. Microplates were incubated at 37 °C for 20 h, and the lowest concentrations of antibacterial agents that completely inhibits visible bacterial growth were determined as MICs.

### 2.6 Genome sequencing and genomic analysis of phage-resistant bacterial strains

Genomic DNA was extracted from *A. baumannii* MRAB11 WT and its phage-resistant strains and sequenced on Oxford Nanopore and Illumina NovaSeq platforms at Personalbio Technology Co., Ltd. (China). Sample preparation and sequencing library construction were performed in accordance with the manufacturer’s protocol. Raw single-molecule sequencing data were assembled using Unicycler (v0.5.0) and Flye (v2.9.1) to generate contigs which were then corrected using NovaSeq high-quality data with Pilon (Walker et al., 2014) (v1.24), followed by final assembly to obtain complete genomic sequences. The genome sequences of WT and phage-resistant strains were analyzed using Mauve (Darling et al., 2004) to assess genomic collinearity, and open reading frames (ORFs) were predicted using GeneMarkS (Besemer et al., 2001; Lomsadze et al., 2018). ORF comparisons were performed using BLASTN (Camacho et al., 2009), according to the information derived from collinear sequence alignments, ORF size, and genomic position. Single nucleotide polymorphisms (SNPs) were identified using GATK (v4.4.0) (McKenna et al., 2010) according to the following procedures. Raw sequencing reads were first aligned to the reference genome using BWA (v0.7.12-r1039) (Li and Durbin, 2009), and subsequent processing was carried out to optimize data quality prior to variant calling. Specifically, duplicates generated during the sequencing were removed using the MarkDuplicates module implemented in Picard (v1.107) (Broad Institute, 2019), with only the paired reads carrying the highest quality scores being retained. Furthermore, to minimize mapping errors and enhance the overall accuracy of SNP calling, the IndelRealigner command from GATK was employed to perform local realignment for all reads located near InDel regions that are prone to inaccurate mapping. Following this sequential pipeline, high-confidence and reliable SNP information was ultimately obtained. Additionally, identification of *A. baumannii* loci for capsular polysaccharide (KL) in genome assemblies with Kaptive (Wick et al., 2018; Wyres et al., 2020). Insertion sequences (IS) and certain transposons were detected through ISfinder (Siguier et al., 2006).

### 2.7 Capsule staining

Capsule staining was performed using the Maneval’s method (Gordillo Altamirano et al., 2021; Hughes. and Smith., 2016). A Congo red drop was placed on a clean glass slide, mixed with 10 μL logarithmic phase bacterial suspension, and spread into a thin smear. After air-drying, the smear was covered with Maneval’s solution for 5 min. Subsequently, the slide was gently lifted and tilted over an elution tray to remove excess stain, rinsed with distilled water, blotted on absorbent paper, and air-dried before oil immersion microscopy.

### 2.8 Phage absorption assay

Phage adsorption assay was conducted according to a previously described method with minor modifications (Gordillo Altamirano et al., 2021). The tested bacteria were cultured to the logarithmic growth phase and adjusted to a concentration of 1×10_7_ CFU/mL. Phages at an MOI of 0.001 were incubated with each tested bacterial strain at 37 °C and 260 rpm for 0, 5, 10, and 20 min. At each time point, 100 μL of the mixture was collected and centrifuged at 16,000×g for 1 min at 4 °C. The supernatant was harvested and filtered through a 0.22−μm filter for quantification of free phage particles. Phage titers were determined using the double-layer agar method. All experiments were performed independently in triplicate.

### 2.9 Scan electron microscopy

Bacteria were cultured to the logarithmic phase, with an OD_600_ of approximately 0.4-0.6. One milliliter of bacterial culture was centrifuged at 10,000 × g for 1 min at 4 °C. The pellet was resuspended in PBS and centrifuged again under the same conditions. After that, the pellet was resuspended in 1 mL of electron microscope fixative (2.5% glutaraldehyde in PBS). Samples were fixed at room temperature in the dark for 30 min and transferred to 4 °C for storage. All samples were transported at 4 °C, and subsequent imaging-related steps were completed by Wuhan Servicebio Technology Co.,Ltd (China).

### 2.10 Transmission electron microscopy

Ten microliters of bacteriophage suspension, purified by CsCl gradient ultracentrifugation (Luong et al., 2020; Wang et al., 2021) and with a concentration of at least 10^10^ PFU/mL, was stored and transported at 4 °C. The samples were then negatively stained with 2% (w/v) potassium phosphotungstate and visualized by Servicebio Technology Co.,Ltd. (China).

### 2.11 Bacterial growth curves

Time-growth curves were determined as previously described (O’Flynn et al., 2006; Sattar et al., 2022; Yuan et al., 2019). Briefly, bacterial cultures in the logarithmic phase were adjusted to 1×10^7^ CFU/mL, incubated at 37 °C, and 0.1 mL aliquots were collected at predetermined time intervals to measure OD_600_. For the phage-treated experiments, MRABP9 (MOI = 0.01) and a phage cocktail comprising MRABP9 (MOI = 0.01) and MRABphi22 (MOI = 1, 0.1, and 0.01) were administered. All experiments were performed independently in triplicate.

### 2.12 Heatmap illustrating phage-mediated lysis and phage resistance

Each tested bacterial strain was cultured in LB broth to mid-log phase and adjusted to a concentration of 5 × 10^8^ CFU/mL. The culture was then divided into four 4-mL aliquots, representing four distinct groups. Fixed doses (10 μL) of phage MRABP9 and/or MRABphi22, each at approximately 1 × 10^9^ PFU, were added to the designated phage-treated groups. The mixtures were incubated, and 0.2 mL aliquots were collected at 0, 4, 10, and 16 h to measure OD_600_. OD_600_ values reflecting bacterial turbidity, were used for generating the heatmap.

### 2.13 Serum sensitivity test

Fresh guinea pig serum was obtained from Berseebio Technology Co., Ltd. (China), with complement activity≥100 U per aliquot. The experimental procedures have been described (Abd El-Aziz et al., 2019; Gordillo Altamirano et al., 2021): normal serum was divided into two equal portions—one remained untreated (NS), while the other was heat-inactivated at 56 °C for 30 min (HS). Both sera were diluted to 50% in PBS. Bacteria (~10^6^ CFUs) in logarithmic phase were inoculated into 500 μL of the 50% serum solution and incubated under 37 °C. Samples were collected every 30 min, and CFUs were determined to assess changes in viable bacterial numbers over time.

### 2.14 Virulence comparison of phage-resistant strains

Various doses of MRAB11 WT were administered intraperitoneally to female BALB/c mice aged 6–8 weeks, and survival was monitored over a 5-day period. Mouse mortality typically occurred within 48 h post-inoculation. A non-lethal dose was selected as the challenge dose, with a bacterial concentration of 2×10^8^ CFU/mL and an injection volume of 0.1 mL per 10 g body weight. Tested phage-resistant strains were cultured to the logarithmic phase, and then administered intraperitoneally at the predetermined challenge dose. The survival of mice was monitored over 5 days.

### 2.15 Statistical analysis

Statistical analyses were performed with GraphPad Prism (v8.0.1). Numbers of biological replicates, statistical tests used and *p* values are specified in figure legends. Wound areas were measured using Fiji software, and the line graph along with statistical analyses were generated using GraphPad Prism. Data are expressed as mean ± standard deviation (SD). Statistical significance was calculated by two-way ANOVA with the Tukey’s multiple comparisons test. Asterisks (*) indicate overall significant differences between groups over the entire observation period, while hash symbols (#) represent significant differences compared with the control group at the corresponding time points. Statistical significance was set at **p < 0.01, ***p < 0.001, #*p* < 0.05 and ##*p* < 0.01. The area under the curve (AUC) of the bacterial growth curves was calculated using GraphPad Prism software. The AUC value of each bacterial strain was treated as one data point, and data are presented as the mean ± SD. The Kruskal-Wallis test and Dunn’s test were used for statistical comparison. *p < 0.05, and ns indicates no statistical difference.

## 3 Results

### 3.1 Isolation of phage-MRABP9-resistant bacterial strains

**Figure 1:**
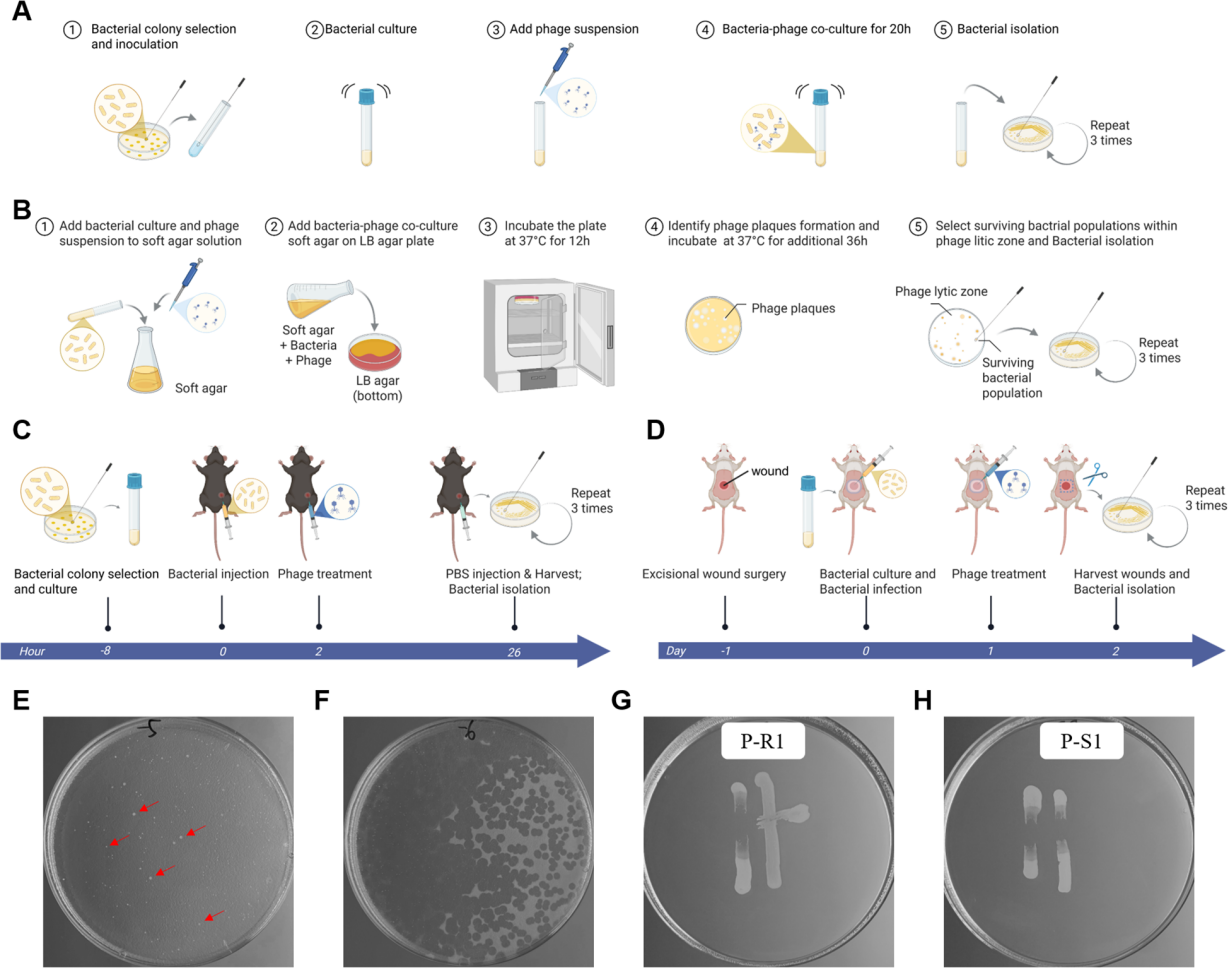
Isolation of phage-resistant bacterial strains. **(A–D)** Phage-resistant bacterial strains were isolated from four distinct sources: liquid culture, plate culture, abdominal infection mouse model, and mouse wound infection model. Created in BioRender. **(E, F)** Double-layer agar plates were prepared using phage stocks diluted 10^5^ times (**E**) and 10_6_ times (**F**). The white regions represent bacterial colonies (red arrows) or remnants of the bacterial lawn following phage-mediated lysis. **(G, H)** Representative images of the cross-streaking method for detecting phage sensitivity. The tested strains were P-R1 **(G)** and P-S1 **(H)**. In the Petri dish, the MRAB11WT appears as a band on the left, with the test strain on the right. Phage MRABP9 was horizontally streaked across the plate, resulting in a broken band for the sensitive strain **(H)**, while the phage-resistant strain exhibited a trailing band extending toward the right **(G)**. Shao, Y. (2026) https://BioRender.com/z7uzsd0.

The antibacterial activities of phage MRABP9 against carbapenem-resistant *A. baumannii* (CRAB) strain MRAB11 have been previously characterized (Zhang et al., 2024). While phage MRABP9 demonstrated strong antibacterial and anti-biofilm properties, phage resistance emerged. Specifically, phage MRABP9 (MOI = 0.01) persistently inhibited host bacterium MRAB11 growth in LB broth for 10 h, and bacterial separation from the phage lysate was performed at 20 h post-co-cultivation (**Figure 1A**). Residual bacteria within the lytic zone after 48-h co-culture were isolated (**Figures 1B, E, F**). Isolation of phage-resistant strains from mouse infection models was also performed (**Figures 1C, D**).

The isolates were tested for phage resistance using the cross-streak method (**Figures 1G, H**). 14 phage-MRABP9-resistant isolates were obtained from liquid cultures, 7 from agar plates, and 5 from mouse CRAB infection models. A total of 15 isolates were randomly selected and subjected to 10 serial passages *in vitro*. All tested isolates remained resistance to phage MRABP9 (**Supplementary Figure 1**), suggesting that the observed phage resistance is genetically stable. Furthermore, these strains were confirmed to originate from the parental strain MRAB11 WT, as verified by whole-genome sequencing (**Table 1**).

**Table 1:** Genetic changes potentially associated with bacteriophage resistance.

Phage-resistant mutants		Mutation type	Mutation description	Related ORFs of WT	Related translational products
*In vitro*	P-R1	Insertion	IS leads to amino acid sequence truncation	ORF3805	ItrA2, initiate capsule biosynthesis
	P-R2	Insertion	IS leads to amino acid sequence truncation	ORF3806	UDP-D-galactose glycosyltransferase Gtr5
	P-R5	Insertion	IS leads to amino acid sequence truncation	ORF3806	Gtr5
	L-R1	Insertion	IS leads to amino acid sequence truncation	ORF3806	Gtr5
	L-R2	Insertion	IS leads to amino acid sequence truncation	ORF3806	Gtr5
	DPR1	SNP	Point mutation, G3966260A	ORF3805	ItrA2
		SNP	Point mutation	ORF545	BaeS, Sensor histidine kinase efflux regulator
	DPR2	SNP	Point mutation, G3966260A	ORF3805	ItrA2
		Deletion	AAA, 3-base loss	ORF545	BaeS
		SNP	Point mutation	ORF545	BaeS
	DPR3	Insertion	IS leads to amino acid sequence truncation	ORF3805	ItrA2
		Insertion	IS leads to amino acid sequence change	ORF3115	Trehalose-6-phosphatase
	DPR4	SNP	Point mutation, G3966260A	ORF3805	ItrA2
		SNP	Point mutation	ORF545	BaeS
*In vivo*	VOS1	Insertion	A single base C insertion, causing frameshift mutation	ORF3802	Glucose-6-phosphate isomerase, responsible for capsule biosynthesis
	VOS3	Insertion	IS leads to amino acid sequence truncation	ORF3805	ItrA2
	VOS4	Insertion	IS leads to amino acid sequence truncation	ORF3805	ItrA2
	VOS6	Deletion	A single base T deletion, causing frameshift mutation	ORF3802	Glucose-6-phosphate isomerase
	SK-2#25	Insertion	IS leads to amino acid sequence truncation	ORF3805	ItrA2
	SK-3#6	Insertion	IS leads to amino acid sequence truncation	ORF3806	Gtr5

### 3.2 Phage MRABP9-resistant mechanisms were highly consistent on capsule synthesis

Given the genetic stability of the phage MRABP9 resistance phenotype, we hypothesize that genomic alterations might occur in resistant strains. We performed whole-genome sequencing of MRAB11 WT and MRABP9-resistant strains, including 9 *in vitro* and 6 *in vivo* isolates, followed by comparative genomic analyses. MRAB11 WT has a 4,054,512-bp chrome genome with 3,892 predicted genes and 39.05% GC content. It also carries an 11,194-bp plasmid encoding 15 predicted genes (**Supplementary Table 1**), and 34.78% GC content. Comparative analysis revealed anomalies in capsular synthesis-related genes in both *in vitro* and *in vivo* resistant strains. Kaptive analysis identified the KL2 capsular genotype in strain MRAB11 (**Supplementary Figure 2**), and all sequenced resistant isolates carried genetic mutations in the KL2 locus, involving in *gtr5*, *itrA2*, and *gpi*.

Among 15 MRABP9-resistant strains including dual-phage-resistant (DPR) strains resistant to both phage MRABP9 and phage MRABphi22 (detailed in Section 3.3 below), eight isolates (P-R1, DPR1, DPR2, DPR3, DPR4, VOS3, VOS4, and SK-2#25) carried *itrA2* mutations involving IS or SNP. *itrA2* located at the KL2 locus encodes an oligosaccharide-initiating transferase that initiates capsular polysaccharide biosynthesis (Kenyon and Hall, 2013; Kenyon et al., 2014) and mutations in this gene may potentially disrupt capsular synthesis. For instance, P-R1 carried an ISAba1 (frequently found in *A. baumannii* genomes (Zhao and Hu, 2012)) homolog (99.7% identity), truncating ItrA2 from 206 to 155 residues. In contrast, *itrA2* remained unaltered in strains P-R2, P-R5, L-R1, L-R2, and SK-3#6, whereas *gtr5* mutations were observed. *gtr5* encodes UDP-D-galactose glycosyltransferase (Gtr5) for oligosaccharide synthesis (Kenyon et al., 2019, Kenyon et al., 2014). All five strains had IS-mediated *gtr5* mutations with varying sites and fragment sizes, indicating transposition randomness. It is noteworthy that *in vivo* resistant strains VOS1 and VOS6 had *gpi* mutations (**Table 1**): frameshifts from single-base deletions/insertions altered glucose-6-phosphate isomerase sequences, causing aberrant capsule synthesis. Other genetic alterations not directly related to phage resistance are detailed in **Supplementary Table 2**.

**Figure 2:**
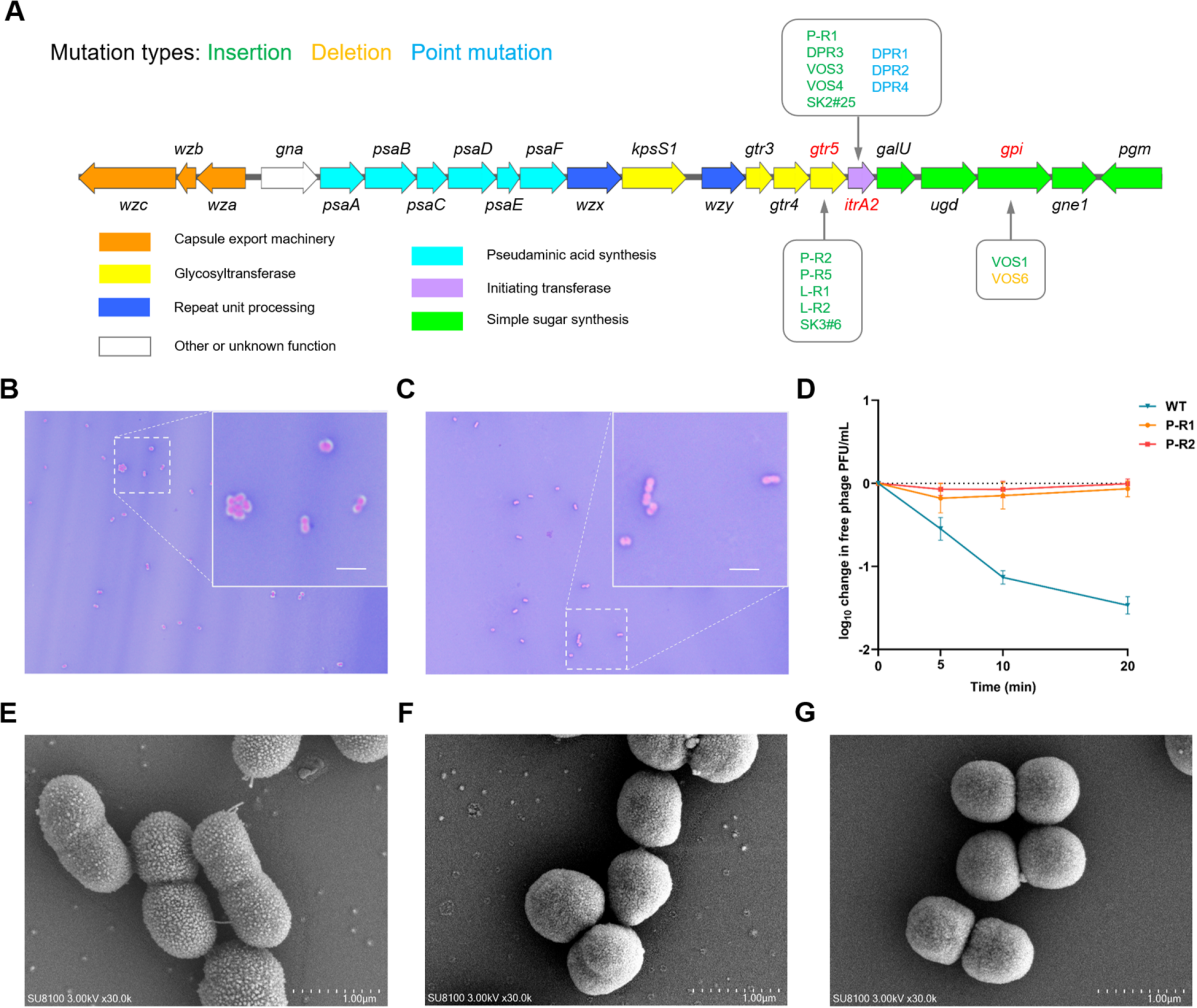
Abnormal capsular synthesis in MRABP9-resistant strains. **(A)** Schematic representation of the capsule synthesis locus KL2 of *Acinetobacter baumannii* strain MRAB11. Arrows indicate the orientation of ORFs, with the red dashed box highlighting the gene mutated in MRABP9-resistant strains. **(B, C)** Capsule staining was carried out on both the WT **(B)** and the phage-resistant strain P-R1 **(C)** using Maneval’s capsule staining method. Scale bar, 2μm. **(D)** Bacteriophage adsorption across distinct bacterial strains. **(E-G)** Morphology of WT **(E)**, phage-resistant strain P-R1 **(F)** and P-R2 **(G)** as observed under scanning electron microscopy. Scale bar, 1μm.

Genomic sequencing revealed all MRABP9-resistant strains harbored mutations in capsule synthesis genes (**Table 1**; **Figure 2A**). Capsule staining (**Figures 2B, C**) and scanning electron microscopy (**Figures 2E–G**) demonstrated aberrant capsule synthesis in resistant strains that could not be adsorbed by phage MRABP9 (**Figure 2D**). Since *Acinetobacter* bacteriophages can utilize the capsule as the adsorption receptor (Gordillo Altamirano et al., 2021; Liu et al., 2022), these findings confirm that KL2 capsule serves as the receptor for MRABP9 infection and capsular aberrations constitute the molecular basis for phage resistance. More importantly, the resistant mechanisms against phage MRABP9 both *in vivo* and *in vitro*, exhibit a high degree of consistency, suggesting that strategies for counteracting resistance evolution may prove effective. This implies the potential existence of a phage capable of broadly targeting MRABP9-resistant bacterial strains.

### 3.3 The bacteriophage MRABphi22 demonstrated effective targeting against MRABP9-resistant isolates

**Figure 3:**
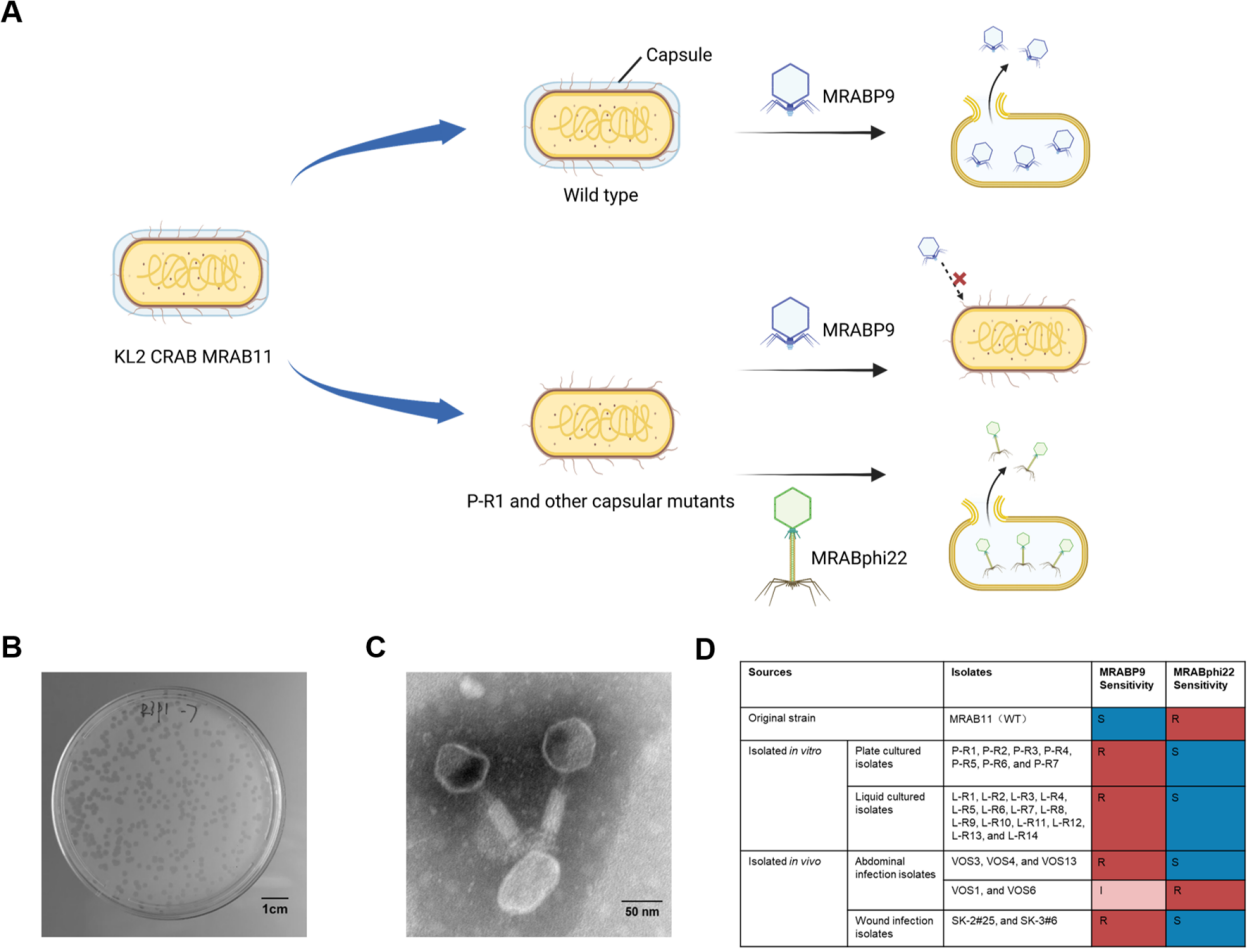
The bacteriophage MRABphi22 effectively targets MRABP9-resistant isolates. **(A)** Schematic illustration of the experimental design. Created in BioRender. **(B)** Phage MRABphi22 forms clear and transparent plaques on the bacterial lawn of P-R1. Scale bar: 1 cm. **(C)** Transmission electron micrograph of phage MRABphi22. Scale bar represents 50 nm. **(D)** Sources and bacteriophage susceptibility of the bacterial strains isolated in this study. Shao, Y. (2026) https://BioRender.com/i5nk5ez.

On the basis of the conserved phage-resistant mechanism, a new round screening of bacteriophage targeting MRABP9-resistant strains were conducted (**Figure 3A**). Accordingly, phage MRABphi22 (GenBank accession number: PQ797121) was isolated from hospital sewage (**Figures 3B, C**). Phage MRABphi22 demonstrated potent lytic activity against all 21 MRABP9-resistant strains derived from *in vitro* sources, as well as 5 out of 7 resistant strains obtained *in vivo*, with the exception of two *gpi*-mutated strains (**Figure 3D**). The presence of the *gpi* mutants suggests that an alternative mechanism may exist, which enables bacterial resistance to both phages, although the exact mechanism remains unclear. Taken together, these findings indicate that MRABphi22 has broad-spectrum targeting capability against MRABP9-resistant isolates, while simultaneously confirming the high degree of consistency in the resistant mechanisms to MRABP9.

### 3.4 The dual-phage cocktail demonstrates high efficacy in controlling CRAB infection and phage resistance

**Figure 4:**
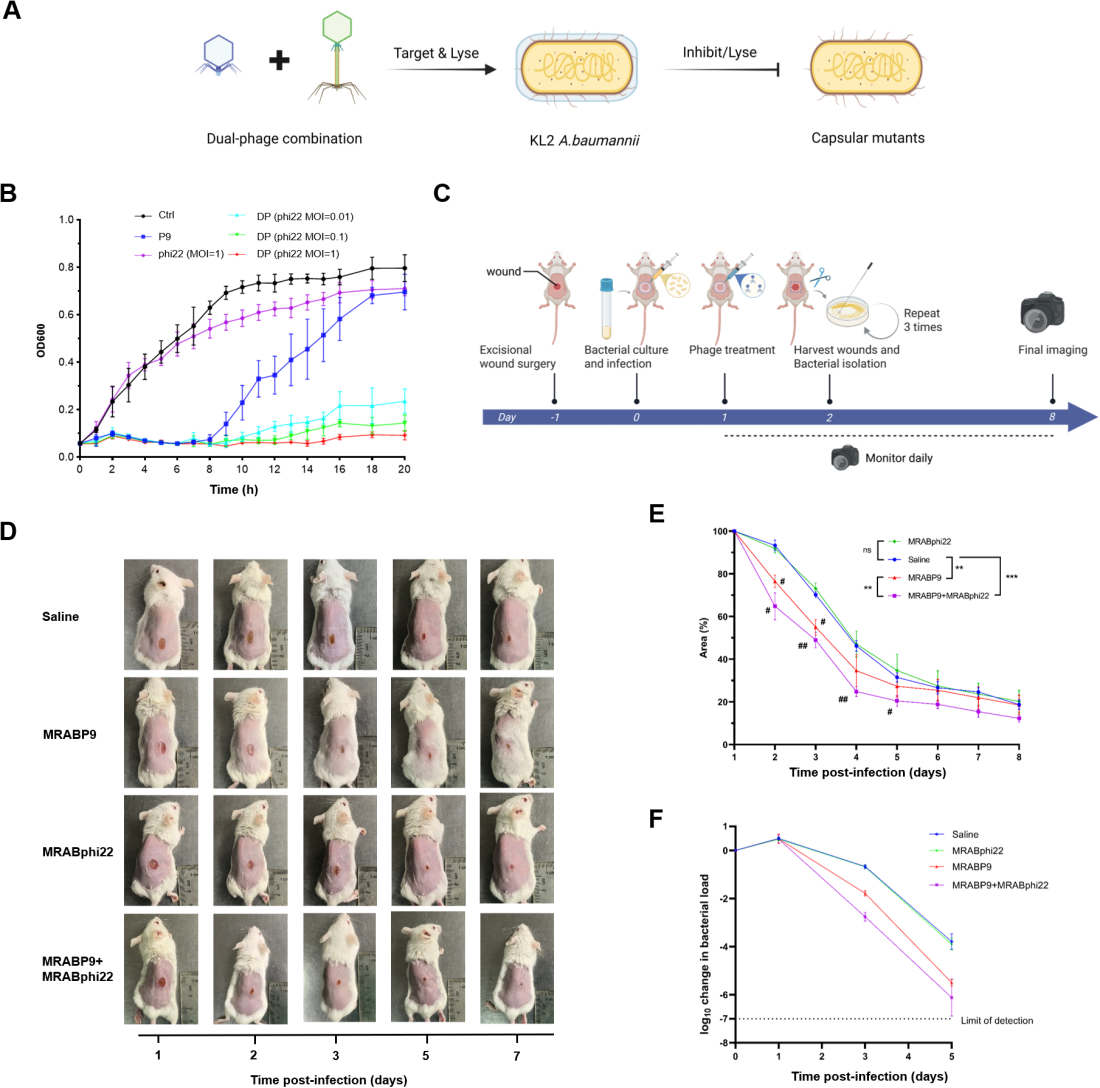
The dual-phage cocktail demonstrates high efficacy in controlling bacterial growth and phage resistance of carbapenem-resistant *A. baumannii*. **(A)** Schematic illustration of the experimental design for the dual-phage cocktail. Created in BioRender. **(B)** The dual-phage cocktail suppressed the growth of the bacterial strain MRAB11 and the development of phage resistance. **(C)** Schematic illustration of the establishment of the mouse CRAB wound infection model and subsequent phage treatment. Created in BioRender. **(D)** Representative photographs depicting wound healing progression in mice over time. **(E)** The percentage of remaining wound area. Data are expressed as mean ± standard deviation. Statistical significance was calculated by two-way ANOVA with the Tukey’s multiple comparisons test. ^**^p < 0.01, ^***^p < 0.001, ^#^*p* < 0.05 and ^##^*p* < 0.01. **(F)** log_10_ change in wound bacterial load following bacterial infection. The initial bacterial load, corresponding to the initial infectious dose, was 10^7^ CFU/wound. Data are expressed as mean ± standard deviation. Shao, Y. (2026) https://BioRender.com/olijgqu; https://BioRender.com/4hfcrwi.

Based on the broad-spectrum antibacterial effect of phage MRABphi22 against MRABP9-resistant isolates, we hypothesized that the combined application of both phages could effectively suppress the growth of the original strain MRAB11 WT and inhibit the progression of phage resistance (**Figure 4A**). The antibacterial effects of the phages were evaluated by culturing the host bacteria with phage MRABphi22 and phage MRABP9 individually, as well as by co-culturing the host bacteria with both phages together (**Figure 4B**; **Supplementary Figure 3**). Phage MRABP9 inhibits bacterial growth for approximately 8 h, after which the growth of resistant strains leads to an increased OD value (blue curve). However, when combined with phage MRABphi22, the antibacterial effect is markedly enhanced. The OD_600_ value remains below 0.2 for up to 16 h, and the inhibitory effect exhibits a dose-dependent trend. In addition, during the middle and later stages of the experiment (8–20 h), certain differences were observed between the normal growth control group and the phage MRABphi22 treatment group (black and purple lines in **Figure 4B**), which may be attributed to the inhibition of a small bacterial subpopulation by phage MRABphi22. Overall, the findings demonstrate that dual-phage cocktail effectively inhibits the growth of the host *A. baumannii* and suppresses the development of phage resistance.

Wound represents one of the most prevalent sources of CRAB isolation, accounting for 20% (Wang et al., 2024). To visually assess the therapeutic efficacy of phage treatment and facilitate bacterial isolation following phage intervention, a mouse model of CRAB wound infection was established (**Figure 4C**). Wound healing was monitored daily starting from the day of phage administration (**Figures 4D, E**). In addition, 3 mice per group were euthanized at 1, 3, and 5 days post-infection for the determination of wound bacterial load (**Figure 4F**). The wound area in the saline-treated group gradually decreased over 7 days (**Figure 4E**, blue line), while wound bacterial load gradually declined by 5 days post−infection (**Figure 4F**, blue line), reflecting natural wound healing and bacterial clearance capacity of mice. In contrast, treatment with phage MRABphi22 did not result in a significant improvement in wound closure within the same period (**Figures 4E, F**). In addition, phage MRABP9 significantly accelerated wound healing within 2 days post-treatment (*p* < 0.05, red line), with a markedly higher healing rate compared to the saline group by day 7. Notably, the wound area in the dual-phage group was significantly smaller than that in saline group within the first 4 days. The healing rate of the cocktail-treated group was significantly faster than that of the saline group (*p* < 0.001), the phage MRABphi22 group (*p* < 0.001), and the phage MRABP9 group (*p* < 0.01), indicating that the dual-phage combination significantly promoted wound healing. Of note, dual-phage treatment led to an apparent reduction in bacterial load within 5 days, albeit with no significant difference relative to the phage MRABP9-treated group. In particular, no viable bacteria were detected in the wound of one mouse from the dual-phage group (**Figure 4F**, purple line). This enhanced effect may be attributed to the role of MRABphi22 in suppressing MRABP9-resistant strains.

### 3.5 Fitness trade-offs occurred in phage-resistant strains, with the DPR strains bearing a higher fitness cost

While the dual-phage strategy has demonstrated promising efficacy in combating CRAB and phage resistance, the existence of dual-phage resistance was still observed (**Figure 4B**). The fitness differences among WT, mono-phage-MRABP9-resistant, and DPR strains have been comparatively analyzed.

**Figure 5:**
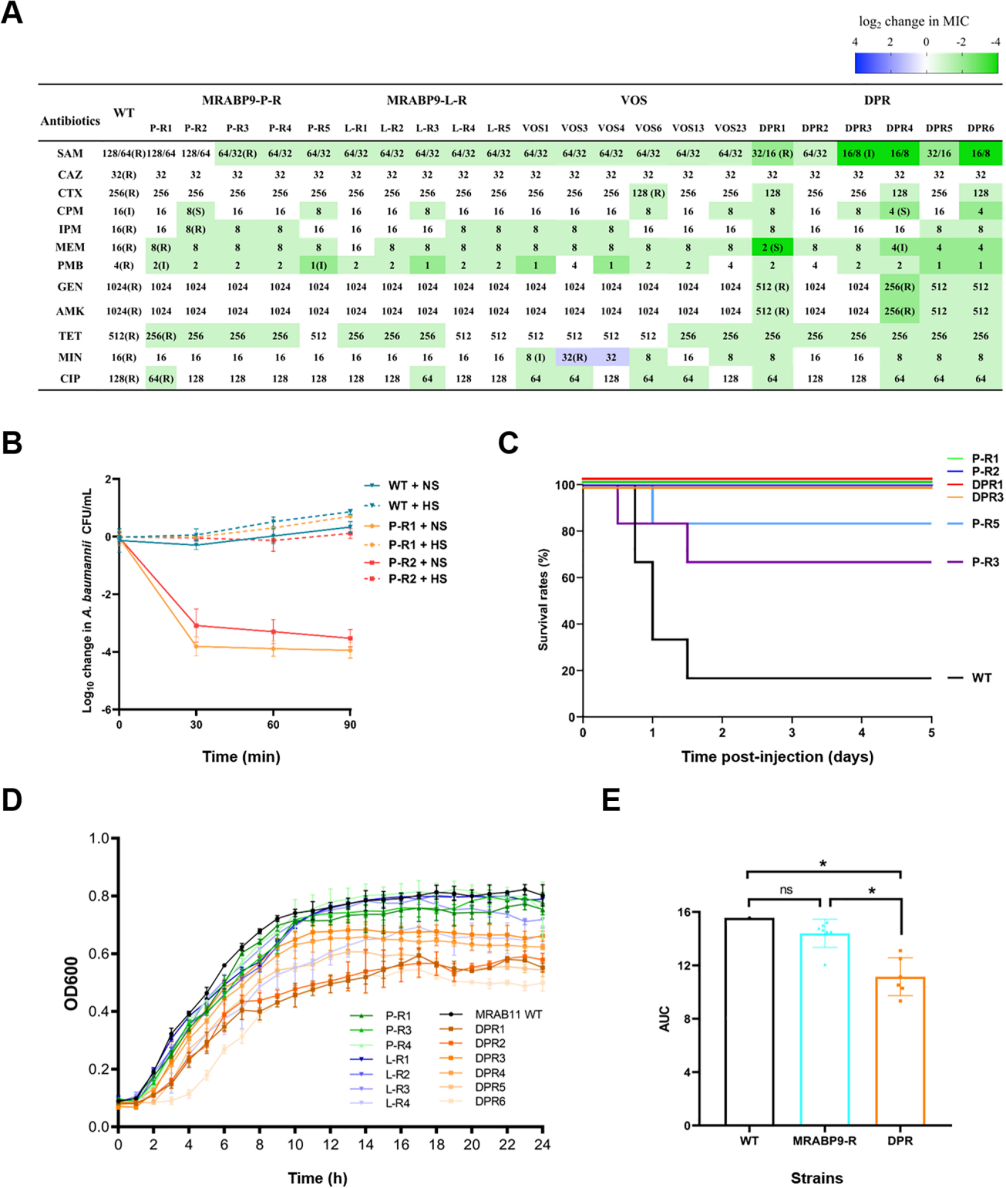
Fitness trade-offs occurred in phage-resistant strains, with the DPR strains bearing a higher fitness cost. **(A)** Determination of MICs (μg/mL) for phage-resistant strains using the broth microdilution method. The abbreviations R (resistant), I (intermediate), and S (susceptible), enclosed in parentheses after the numerical MIC values, indicate interpretive category of antimicrobial susceptibility based on the 33th edition of CLSI M100. SAM, ampicillin with sulbactam; CAZ, ceftazidime; CTX, cefotaxime; CPM, cefepime; IPM, imipenem; MEM, meropenem; PMB, polymyxin B; GEN, gentamicin; AMK, amikacin; TET, tetracycline; MIN, minocycline; CIP, ciprofloxacin. **(B)** Assessment of bacterial susceptibility to serum. NS, normal serum; HS, heat-inactivated serum. **(C)** Survival curves of mice following injection with equivalent doses of different strains. **(D)** Growth curve of MRABP9-resistant strains. The black curve represents MRAB11 WT, while the varying shades of green curves denote MRABP9-P-R, the differing intensities of blue curves indicate MRABP9-L-R, and the graded orange curves correspond to DPR. Each bacterial strain was assayed in triplicate. **(E)** Comparison of the area under the curve (AUC) for bacterial growth curves derived from **(D)**. WT refers to the wild-type MRAB11, with only 1 bacterial strain, n = 1; MRABP9-R represents the phage MRABP9-resistant strains, including P-R and L-R, n = 7; DPR stands for the dual-phage resistant strains, n = 6. The Kruskal-Wallis test and Dunn’s test were used for statistical comparison. *p < 0.05, and ns indicates no statistical difference. Created in BioRender. Shao, Y. (2026) https://BioRender.com/qdblrnh.

Antibiotic resistance of phage-resistant strains to 12 antibiotics across seven major categories was evaluated (**Figure 5A**). Mono-phage-MRABP9-resistant strains (including MRABP9-P-R, MRABP9-L-R, and VOS) exhibited reduced resistance to certain antibiotics. Specifically, among the 16 tested mono-phage-MRABP9-resistant strains, 15 exhibited reduced MIC of meropenem and 14 showed decreased MIC to ampicillin-sulbactam, while the interpretive category remained resistant; 14 displayed diminished resistance to polymyxin B, with a shift from R to I. In addition, the MICs of some isolates to cefepime, imipenem, tetracycline, and ciprofloxacin were decreased, suggesting increased antibiotic susceptibility. Notably, MICs of all mono-phage-MRABP9-resistant strains to ceftazidime, gentamicin, and amikacin remained unchanged. It is noteworthy that *in vivo* phage-resistant isolates VOS3 and VOS4 exhibit elevated MICs to minocycline, a tetracycline-class antibiotic. Combined with the results of comparative genomics analysis (**Supplementary Table 2**), a large fragment insertion was identified in VOS3, which harbors a gene (*ORF3784*) encoding Tet(B), a tetracycline efflux transporter belonging to the MFS family (Beheshti et al., 2020; Roberts, 2005). Since minocycline is a tetracycline-class antibiotic (Beheshti et al., 2020; Chopra and Roberts, 2001), this insertion is speculated to be the key factor responsible for the altered minocycline MIC. Nevertheless, VOS4 was unable to detect any genetic mutations specifically associated with minocycline resistance. Therefore, the mechanism responsible for the altered minocycline resistance remains elusive and the development of phage resistance is not necessarily accompanied by a weakening of drug resistance, suggesting that clinical phage applications require careful consideration of antibiotic types and their potential interactions with phages. Compared to WT, DPR strains showed a substantial reduction in resistance to nearly all tested antibiotics (**Figure 5A**). DPR strains exhibited even lower levels of antibiotic resistance than mono-phage-MRABP9-resistant strains (**Figure 5A**). Specifically, the resistance of DPR3, DPR4, and DPR6 to ampicillin with sulbactam shifted from R to I; similarly, DPR4, DPR5, and DPR6 exhibited a shift from R to I for meropenem, while DPR1 became susceptible to meropenem. Furthermore, the MICs of gentamicin and amikacin against certain DPR strains were 4-fold lower than both the WT and mono-phage-MRABP9-resistant strains. These indicate that the antibiotic resistance of DPR strains was further diminished, with some strains’ re-sensitive to certain antibiotics, thereby verifying the feasibility of combining phages with antibiotics.

Serum sensitivity and virulence of WT and phage-resistant strains were accessed (**Figures 5B, C**). When treated with heat-inactivated serum, viable counts of all strains remained stable within 90 min. WT maintained population stability even in the presence of normal active serum, attributable to the protective role of its capsule. However, the phage-resistant strains exhibited high sensitivity to normal serum, with approximately 4-log bacterial reduction within 90 min, which indicates a compromised capacity to evade complement-mediated killing. Biofilm formation capacity of phage-resistant strains was also weakened (**Supplementary Figure 4**). Besides, when mice were infected with an identical bacterial dose, lethality rates were 83.3% (*n* = 6) for WT, 33.3% (*n* = 6) for P-R3, and 16.7% (*n* = 6) for P-R5, whereas all mice infected with the remaining strains survived (**Figure 5C**). Across all mono-MRABP9-resistant strains, the overall mortality rate was 12.5% (*n* = 24), while no mortality (0%, *n* = 12) was observed in mice infected with DPR strains. These results demonstrate that resistance to phage MRABP9 is associated with a marked reduction in bacterial virulence, and dual-phage resistance leads to a greater attenuation of pathogenicity.

Furthermore, we compared the growth rates of phage-resistant strains and WT (**Figures 5D, E**). The proliferation rate of MRABP9-resistant strains had a marginal reduction compared to the WT, with no statistical significance, suggesting that acquisition of mono-MRABP9 resistance does not substantially impair bacterial growth (**Figure 5E**, blue column). In contrast, DPR strains exhibited significantly reduced growth rates, with their growth curve AUC markedly lower than both the WT and mono-phage-MRABP9-resistant strains (**Figure 5E**, orange column). Together, these results suggest that while dual-phage application may still encounter challenges related to phage resistance, the fitness of DPR strains has been markedly declined. This reduction provides opportunities and time for antibiotic treatments and the hosts’ immune responses, further supporting the clinical potential of the dual-phage cocktail.

### 3.6 The dual-phage cocktail effectively controlled the bacterial growth and phage resistance of clinical A. baumannii isolates

**Figure 6:**
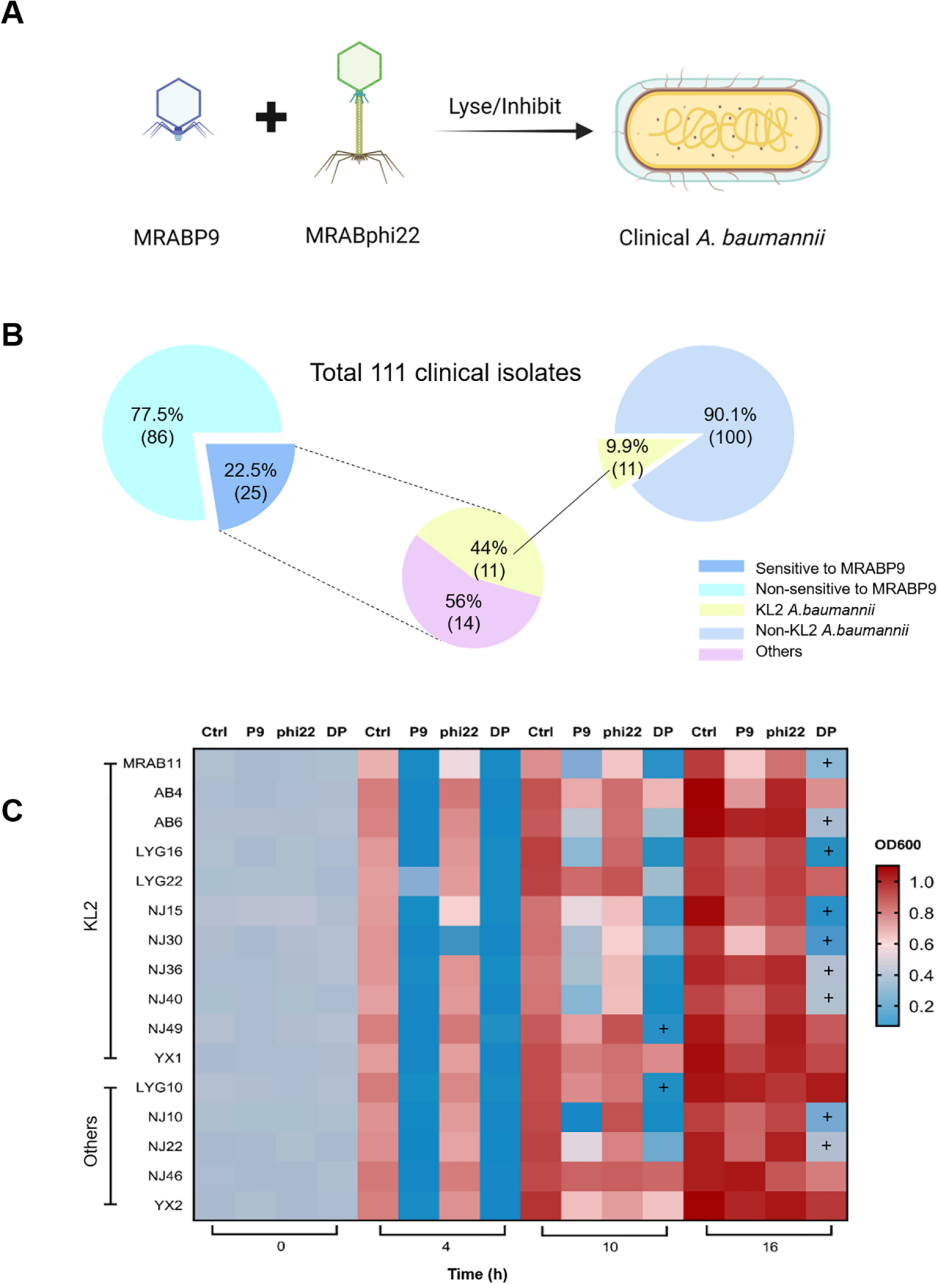
The dual-phage cocktail effectively controlled the growth and phage resistance development of clinical *A. baumannii* isolates. **(A)** Schematic representation of the dual phage combination strategy. **(B)** A total of 111 clinical *A. baumannii* isolates were classified using two distinct approaches: phage MRABP9 sensitivity and capsule type. Notably, all 11 KL2 strains exhibited susceptibility to phage MRABP9. **(C)** The dual-phage cocktail demonstrates efficacy against K2-type and other *A. baumannii* strains. Deeper red hues in the heatmap indicate higher bacterial turbidity, while darker blue shades signify lower bacterial turbidity. Ctrl: Control group, reflecting the normal growth of MRAB11; P9: Treatment group with phage MRABP9; phi22: Treatment group with phage MRABphi22; DP: Co-treatment group with MRABP9 and MRABphi22. “+” indicates enhanced antibacterial efficacy of dual−phage treatment, defined as OD_600_ < 0.5 and OD_600_ difference > 0.2 relative to single−phage treatment. The experiment was performed in triplicate.

Subsequently, we endeavored to determine whether this dual-phage strategy demonstrates efficacy against other clinical *A. baumannii* strains (**Figure 6A**). The host range of MRABP9 was initially assessed (**Figure 6B**). KL2 was the most common KL type of *A. baumannii* (Hsieh et al., 2020; Wyres et al., 2020) and is frequently associated with high antimicrobial resistance and high pathogenicity (Hsieh et al., 2020; Yang et al., 2022). Among the 111 clinical *A. baumannii* isolates, the capsular type of 11 tested isolates was KL2, account for 9.9%. Notably, MRABP9 was capable of lysing 100% KL2 strains (11/11, **Figure 6B**). An additional 14 non-KL2 isolates were susceptible to phage MRABP9, with a total lytic rate of 22.5% (25/111).

We evaluated the efficacy of dual-phage cocktail in 16 clinical isolates sensitive to MRABP9 (**Figure 6C**). The cocktail demonstrated efficacy in 9 out of 11 KL2 isolates, exhibiting a stronger bacteriostatic effect compared to mono-phage MRABP9 treatment. Moreover, the favorable effect of dual-phage combination was also observed in 3 out of 5 non-KL2 isolates. These results indicate that the dual-phage cocktail exhibits a potent and long-lasting antibacterial effect against various KL2 and certain non-KL2 strains, suggesting its broad applicability. The phage combination of MRABphi22 and MRABP9 is anticipated to demonstrate significant efficacy in clinical treatments of KL2 *A. baumannii* infections, while also reducing the risk of infection recurrence due to phage resistance.

## 4 Discussion

Phage therapy holds promise for addressing multidrug-resistant bacterial infections. However, the development of phage resistance may lead to bacterial regrowth and recurrence of infection following phage treatment (Ashworth et al., 2024; Chan et al., 2018; Liu et al., 2022; Schooley et al., 2017; Torres-Barceló, 2018), thereby compromising therapeutic efficacy. This study involved phage-resistant variants that emerged during lysis of CRAB strain MRAB11 by phage MRABP9 and phage-resistant mechanisms were highly consistent both *in vitro* and *in vivo*, as all sequenced phage-resistant isolates harbored mutations in KL2 locus. In addition, MRABP9-resistant strains possess defective capsules that were poorly adsorbed by phage MRABP9, and capsule has been identified as phage receptors of certain *A. baumannii* strains (Deng et al., 2025; Gordillo Altamirano et al., 2021; Liu et al., 2022; Popova et al., 2017; Timoshina et al., 2023), suggesting that mutations in capsule-synthesis-related genes leading to the phage resistance. It was due to the high consistency of *in vitro* and *in vivo* phage-resistant mechanisms that a novel phage MRABphi22 was introduced. Phage MRABphi22 can effectively target a wide range of MRABP9-resistant strains, which further supported the concordance between *in vitro* and *in vivo* phage-resistant mechanisms. This observation aligns with the findings of Liu et al (Liu et al., 2022), suggesting that *in vitro* experimental studies can reliably reflect *in vivo* scenarios, thereby bolstering the translational relevance of conclusions derived from *in vitro* phage experiments to *in vivo* applications.

Phage MRABP9 demonstrated extensive lytic activity against *A. baumannii* clinical isolates (25/111), with a no**Table 1**00% lytic efficacy among the tested KL2 isolates (11/11), indicating its potential as a candidate for clinical intervention against *A. baumannii* infections. Given the efficacy and broad-spectrum activity against MRABP9-resistant isolates, the combination of phage MRABphi22 with MRABP9 was performed and demonstrated to effectively inhibit the growth of the carbapenem-resistant *A. baumannii* MRAB11, while also delaying the development of phage resistance. Additionally, the application of the dual-phage combination in the wound CRAB infection model yielded promising results, suggesting its potential in managing CRAB infections and mitigating the development of phage resistance. Phage cocktails typically incorporate three or more phages to expand the host spectrum and mitigate phage resistance (Forti et al., 2018; Gao et al., 2022; Grygorcewicz et al., 2021; Koncz et al., 2024; Wang et al., 2022b). This study demonstrates that a single phage effectively targets 100% of the tested KL2 clinical isolates, while a dual-phage cocktail sustainably inhibits phage resistance development in KL2 and other clinical *A. baumannii* isolates, supporting its consideration as first-line in clinical phage therapy.

In alignment with prior research findings (Burmeister et al., 2020; Chen et al., 2024; Gao et al., 2022; Gordillo Altamirano et al., 2021; Li et al., 2023, Li et al., 2022), the fitness of MRABP9-resistant strains was attenuated, including antibiotic resistance, immune evasion capacity, and virulence. Importantly, dual-phage-resistant strains exhibited further reductions in antibiotic resistance, virulence, and growth rate. These indicate that bacterial fitness is generally compromised upon acquiring phage resistance, and the cost is higher under dual-phage pressure. Although phage resistance may emerge during therapeutic application, the resultant resistant strains exhibit substantially diminished pathogenicity and resilience, thereby creating a favorable window for immune clearance and complementary interventions. It is worth noting that, certain MRABP9-resistant strains exhibited elevated resistance to minocycline, which is consistent with previously reported increases in MIC values linked to phage resistance. For instance, phage-resistant mutants of *A. baumannii* A9844 showed enhanced resistance to amikacin (Gordillo Altamirano et al., 2021), and mutants of *P. aeruginosa* ZS-PA-35 resistant to phage phiPA10 exhibited increased MICs against meropenem (Li et al., 2022). Collectively, these findings indicate that phage resistance does not necessarily incur a fitness cost in bacterial pathogens, highlighting the need for case-specific evaluations of phage-antibiotic combinations.

Although our study covered 111 clinical isolates from three Chinese cities, extrapolating definitive global conclusions remains challenging. Considering the worldwide prevalence and conservation of KL2, this investigation offers a valuable reference for the global management of KL2 *A. baumannii*. Regarding the underlying mechanism of dual-phage resistance, we identified SNPs in the *baeS* gene across three sequenced DPR strains. Single-base mutations in *baeS* modulated the expression of membrane molecules in *A. baumannii* (Chen et al., 2023; Liu et al., 2023), implying that dual-phage resistance could be associated with alterations in the bacterial membrane mediated by *baeS*. CRAB strain MRAB11 exhibited extensive and high-level drug resistance, and no effective antibiotics or corresponding antibiotic resistance markers were available for genetic screening. Although the m−Cherry fluorescent plasmid was also tested, MRAB11 appeared to exhibit exclusion of exogenous plasmids, likely due to the presence of a native high−copy plasmid (Blackwell and Hall, 2019; Koong et al., 2025; Nang et al., 2019; Salgado-Camargo et al., 2020). Accordingly, the construction of gene knockout and complementation strains was not successfully achieved in the present study. Deciphering the dual-phage resistance mechanism putatively associated with *baeS* will be the focus of our future work.

## 5 Conclusions

The mechanisms mediating the response of CRAB strain MRAB11 to phage MRABP9 infection exhibited considerable consistency. The combined application of a second phage targeting phage-resistant strains together with the original phage demonstrates improved efficacy in combating CRAB infection and phage resistance. This dual-phage therapeutic approach presents a promising clinical candidate for the treatment of *A. baumannii* infections, while simultaneously mitigating the effects of phage resistance. Further investigations should validate these findings and explore extending this dual-phage strategy to other bacterial infections.

## Data Availability

The datasets presented in this study can be found in online repositories. The names of the repository/repositories and accession number(s) can be found in the article/**Supplementary Material**.
