# Supplementary material for: A conserved KL2-capsule-related phage-resistant mechanism in carbapenem-resistant Acinetobacter baumannii is surmountable by a rationally selected dual-phage cocktail
Source: Front Cell Infect Microbiol. 2026 Apr 2;16:1790430. doi: 10.3389/fcimb.2026.1790430 (PMC13083202; doi:10.3389/fcimb.2026.1790430)
Supplement: Supplementary file 1 [file Table1.docx]

Supplementary Material

**
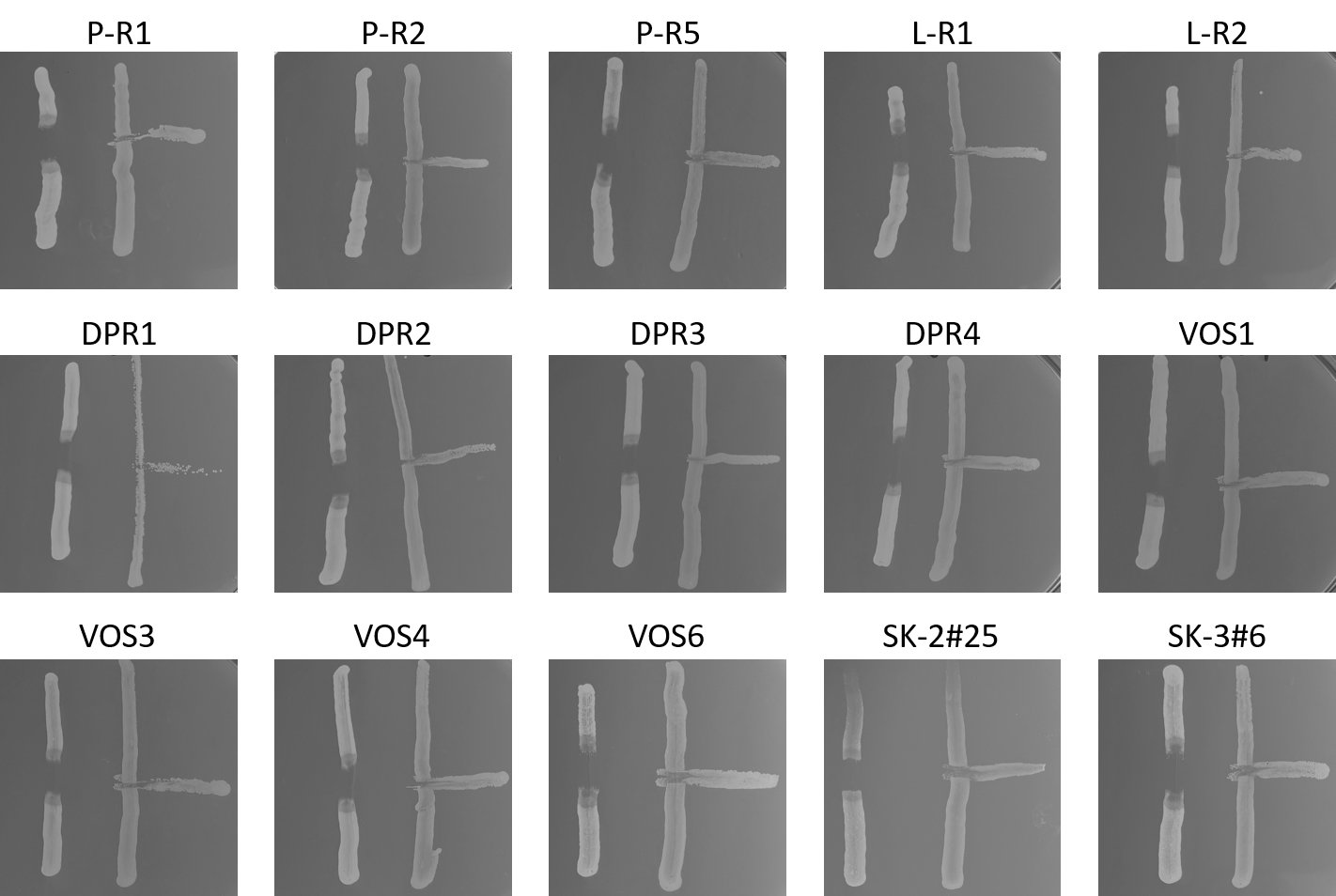
**

**Supplementary Figure 1. The phage resistance of 15 MRABP9-resistant strains was determined by the cross-streak method after 10 serial passages.** The left streak represents the MRAB11 WT, while the right band corresponds to the tested strain.

**
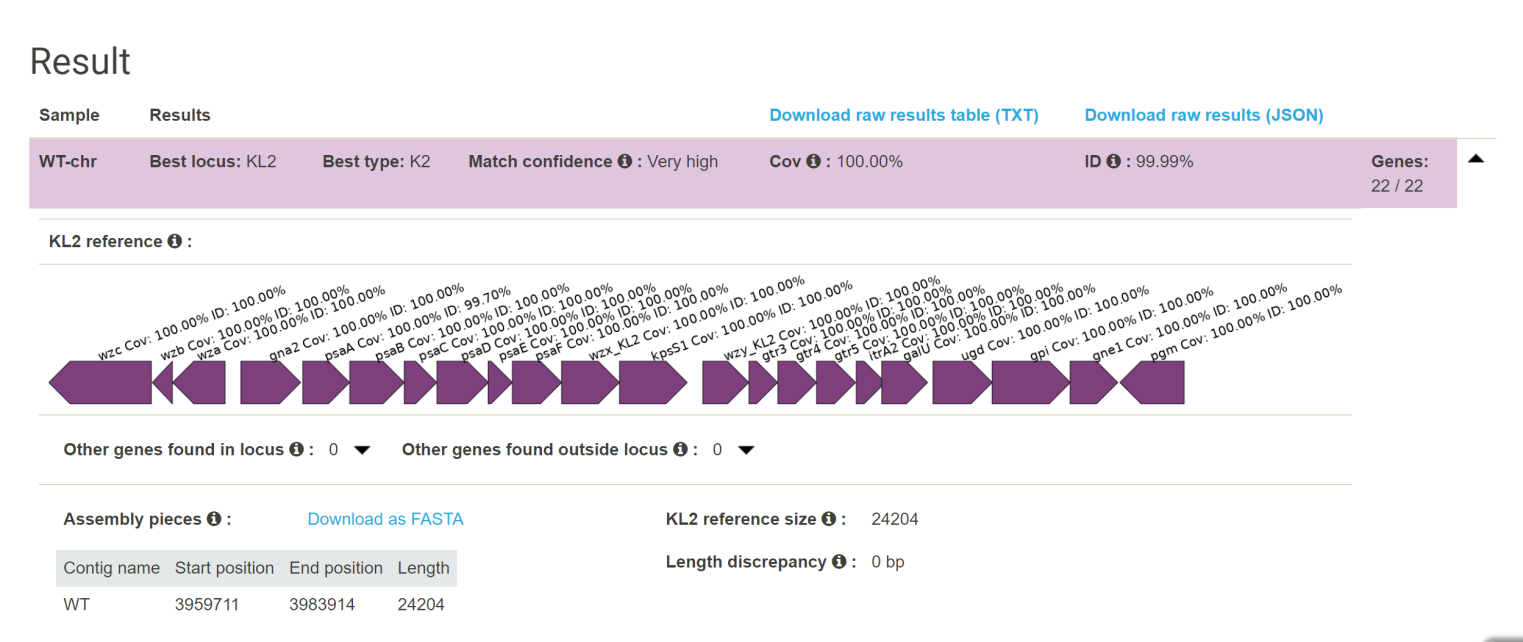
**

**Supplementary Figure 2. KL type identification of carbapenem-resistant *A. baumannii* MRAB11 WT by Kaptive.** MRAB11 WT showed a high degree of matching with the reference sequence of the KL2 locus, with 100% sequence coverage and 99.99% sequence identity.


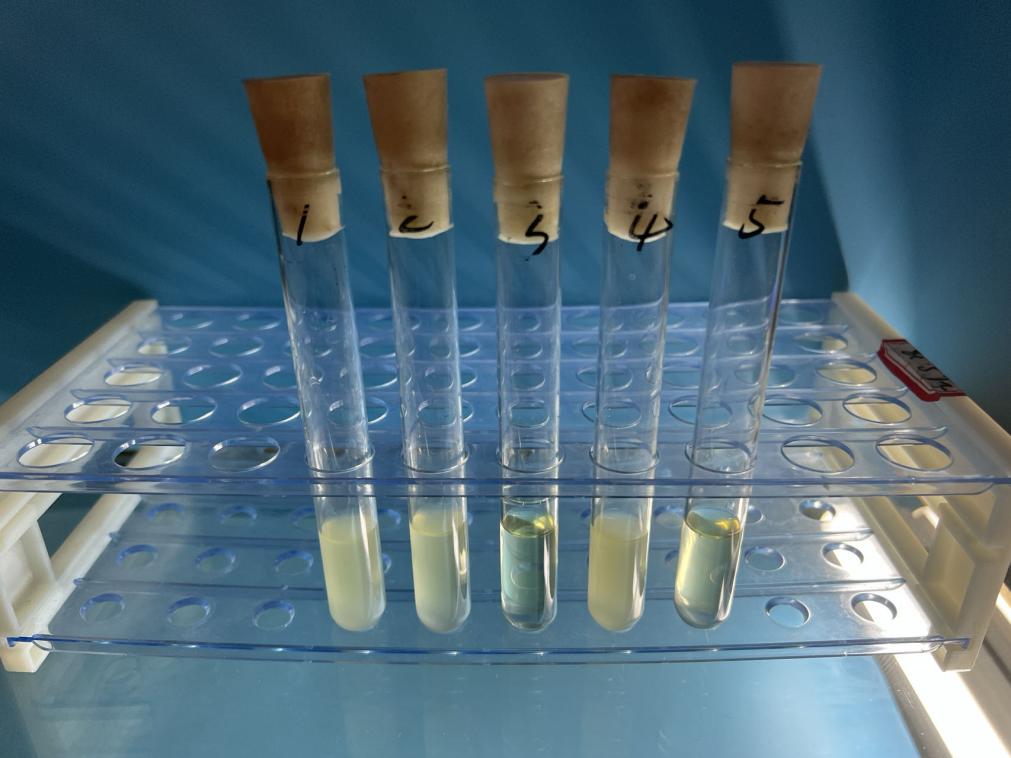


**Supplementary Figure 3. The dual-phage combination suppressed the growth of the bacterial strain MRAB11 and the development of phage resistance.** From left to right, the five test tubes are sequentially labeled as MRAB11, MRAB11 + MRABP9 (MOI=0.01), MRAB11+MRABP9 (MOI=0.01) +MRABphi22 (MOI=1), MRAB11+MRABphi22 (MOI=1), and MRAB11+MRABP9 (MOI=0.01) + MRABphi22 (MOI=0.1).

**
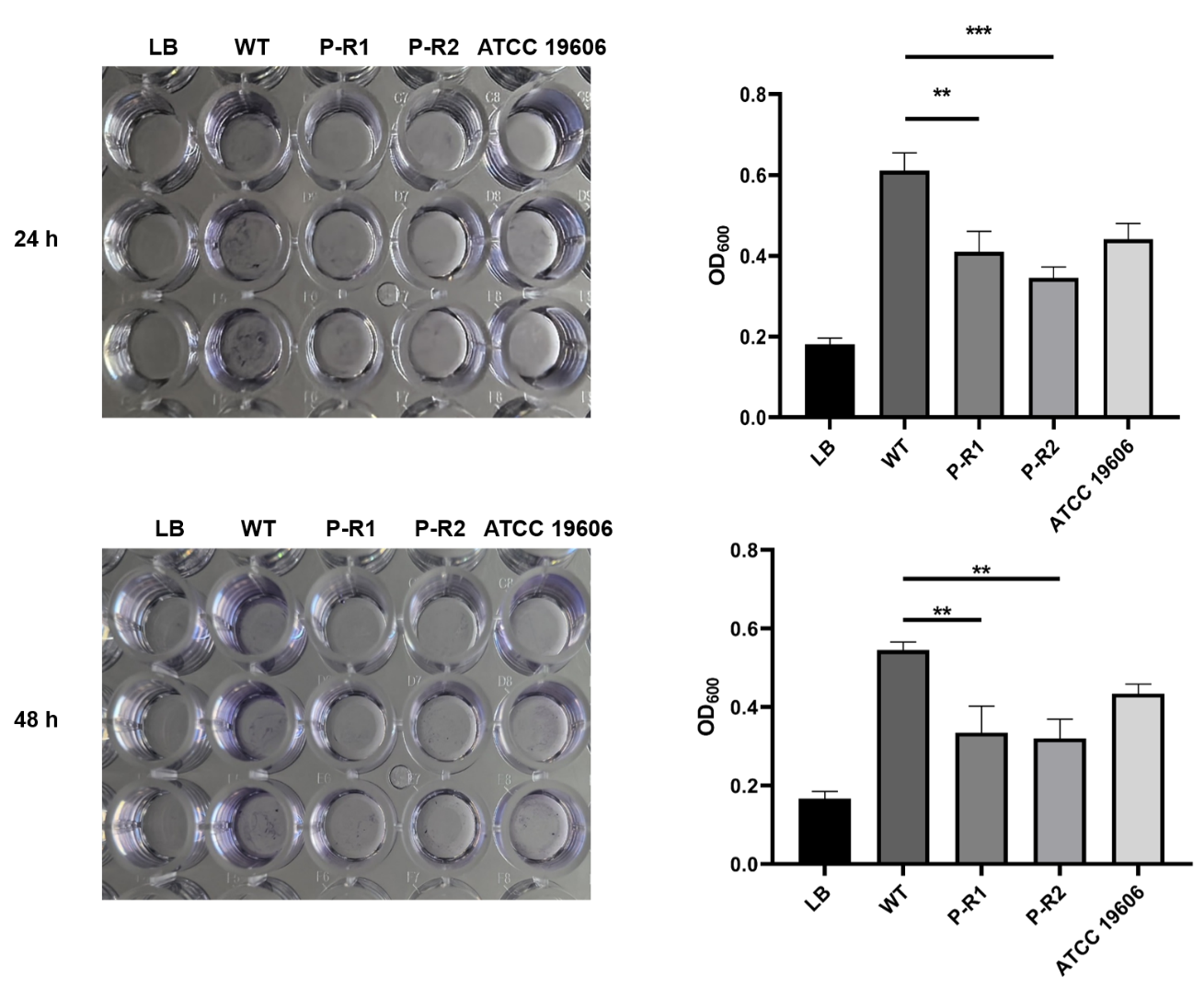
**

**Supplementary Figure 4. Comparison of biofilm formation ability between phage-resistant strains and wild-type strain at 24 h (upper) and 48 h (lower).** The left panel illustrates the biofilm formation outcomes obtained through the crystal violet staining, while the right panel displays the corresponding statistical analysis results. WT refers to MRAB11 WT, while P‑R1 and P‑R2 are phage‑resistant strains resistant to phage MRABP9. An LB group without biofilm formation and the standard strain ATCC19606 were included as negative and positive controls for biofilm formation, respectively. Student’s t-test, **p*<0.05, ***p*<0.01, and ****p*<0.001.

**Supplementary Table 1. Genetic changes identified in phage-resistant mutants of carbapenem-resistant *A. baumannii* MRAB11.**

| **ORFs** | **Strand** | **Nucleotide position** | **Amino acid length (aa)** | **Gene annotation** |
| --- | --- | --- | --- | --- |
| ORF1 | + | 1-462 | 153 | DIP1984 family protein septicolysin |
| ORF2 | - | 788-997 | 69 | hypothetical protein |
| ORF3 | - | 990-129 | 100 | XRE family transcriptional regulator |
| ORF4 | - | 1285-1641 | 118 | type II toxin-antitoxin system RelE/ParE family toxin |
| ORF5 | + | 1689-1793 | 34 | hypothetical protein |
| ORF6 | - | 2207-2506 | 99 | hypothetical protein |
| ORF7 | - | 2623-3495 | 172 | hypothetical protein |
| ORF8 | - | 3647-3814 | 311 | hypothetical protein |
| ORF9 | + | 3833-4858 | 341 | MobA/MobL family protein |
| ORF10 | + | 5455-6390 | 55 | replication initiation protein RepM |
| ORF11 | + | 6468-6986 | 290 | plasmid replication DNA-binding protein |
| ORF12 | + | 7196-7495 | 99 | hypothetical protein |
| ORF13 | + | 7763-8050 | 95 | BrnT family toxin |
| ORF14 | + | 8043-8351 | 102 | BrnA antitoxin family protein |
| ORF15 | + | 8479-10890 | 803 | TonB-dependent receptor ZnuD2 |

**Supplementary Table 2. Genetic changes identified in phage-resistant mutants of carbapenem-resistant *A. baumannii* MRAB11.**

| **Phage-resistant mutants** | | **Mutation type** | **Mutation description** | **Related ORFs of WT** | **Related translational products** |
| --- | --- | --- | --- | --- | --- |
| *In vitro* | P-R1 | Insertion | IS leads to amino acid sequence truncation | ORF530 | LOS outer core biosynthesis glycosyltransferase GtrOC6 |
|  |  | Deletion | Partial loss of repeated sequences | ORF2046 | RTX toxins-related Ca^2+^-binding protein |
|  |  | Insertion | IS leads to amino acid sequence truncation | ORF3805 | ItrA2, initiate capsule biosynthesis |
|  | P-R2 | Insertion | IS leads to amino acid sequence truncation | ORF530 | GtrOC6 |
|  |  | Insertion | IS leads to amino acid sequence truncation | ORF3806 | UDP-D-galactose glycosyltransferase Gtr5 |
|  | P-R5 | Deletion | Large fragment deletion | ORF2908-ORF2917 | Contains DNA helicase, ATPase β lactase, and transposase |
|  |  | Insertion | IS leads to amino acid sequence truncation | ORF3806 | Gtr5 |
|  | L-R1 | Insertion | IS leads to amino acid sequence truncation | ORF530 | GtrOC6 |
|  |  | Deletion | Large fragment deletion | ORF2841-ORF2850 | Contains DNA helicase, ATPase β lactase, and transposase |
|  |  | Insertion | IS leads to amino acid sequence truncation | ORF3806 | Gtr5 |
|  | L-R2 | Insertion | IS leads to amino acid sequence truncation | ORF530 | GtrOC6 |
|  |  | Deletion | Large fragment deletion | ORF2906-ORF2907 | IS element ISAba1 loss |
|  |  | Deletion | Single base loss, frameshift mutation | ORF3049 | Tyrosyl-tRNA synthetase |
|  |  | Insertion | IS leads to amino acid sequence truncation | ORF3806 | Gtr5 |
|  | DPR1 | SNP | Point mutation | ORF545 | Sensor histidine kinase efflux regulator BaeS |
|  |  | SNP | Point mutation, G3966260A | ORF3805 | ItrA2 |
|  | DPR2 | SNP | Point mutation | ORF545 | BaeS |
|  |  | Deletion | AAA, 3-base loss | ORF545 | BaeS |
|  |  | SNP | Point mutation, G3966260A | ORF3805 | ItrA2 |
|  | DPR3 | Insertion | 5-base insertion, frameshift mutation | ORF3049 | Tyrosyl-tRNA synthetase |
|  |  | Insertion | IS leads to amino acid sequence change | ORF3115 | Trehalose-6-phosphatase |
|  |  | Insertion | IS leads to amino acid sequence truncation | ORF3805 | ItrA2 |
|  | DPR4 | SNP | Point mutation | ORF545 | BaeS |
|  |  | Deletion | Large fragment deletion | ORF2908-ORF2917 | Contains DNA helicase, ATPase β lactase and transposase |
|  |  | SNP | Point mutation, G3966260A | ORF3805 | ItrA2 |
| *In vivo* | VOS3 | Insertion | IS leads to amino acid sequence truncation | ORF529 | LPS biosynthesis glycosyltransferase |
|  |  | Insertion | Large insertion mediated by two transposable elements | ORF3805-ORF3806 | Transposases, tetracycline resistance protein, aminoglycoside phosphotransferase and universal stress protein |
|  |  | Insertion | IS leads to amino acid sequence truncation | ORF3805 | ItrA2 |
|  | VOS4 | Insertion | IS leads to amino acid sequence truncation | ORF529 | LPS biosynthesis glycosyltransferase |
|  |  | Deletion | 3-base insertion, amino acid sequence truncation | ORF3650-3651 | StrA, affects resistance to streptomycin |
|  |  | Insertion | IS leads to amino acid sequence truncation | ORF3805 | ItrA2 |
|  | VOS1 | Deletion | Large fragment deletion | ORF2908-ORF2917 | Contains DNA helicase, ATPase β lactase and transposase |
|  |  | Insertion | A single base C insertion, causing frameshift mutation | ORF3802 | Glucose-6-phosphate isomerase, responsible for capsule biosynthesis |
|  |  |  |  |  |  |
|  | VOS6 | Deletion | A single base T deletion, causing frameshift mutation | ORF3802 | Glucose-6-phosphate isomerase |
|  |  | Deletion | Large fragment deletion | ORF2841-ORF2850 | Contains DNA helicase, ATPase β lactase and transposase |
|  | SK-2#25 | Insertion | IS leads to amino acid sequence truncation | ORF529 | LPS biosynthesis glycosyltransferase |
|  |  | Deletion | 2-base deletion, amino acid sequence truncation | ORF2917 | IS4 family transposase |
|  |  | Insertion | Mediated by two transposable elements | ORF3805-ORF3806 | Transposases, tetracycline resistance protein, aminoglycoside phosphotransferase and universal stress protein |
|  |  | Insertion | IS leads to amino acid sequence truncation | ORF3805 | ItrA2 |
|  | SK-3#6 | Insertion | IS leads to amino acid sequence truncation | ORF3806 | Gtr5 |

All changes are relative to the sequence of *A. baumannii* strain MRAB11 WT. Exclusively mutations inducing alterations in the coding sequence are presented, whereas synonymous SNPs and mutations located in non-coding regions have been systematically excluded.
